# Supplementary material for: Mapping the evolution of fertility support policies in China: A content and instrumental analysis
Source: PLoS One. 2025 Oct 9;20(10):e0332137. doi: 10.1371/journal.pone.0332137 (PMC12510515; doi:10.1371/journal.pone.0332137)
Supplement: S1 Appendix — (ZIP) [file pone.0332137.s001.zip › S1 Appendix. 226 original policy documents/79-国务院关于印发“十三五”卫生与健康规划的通知(FBM-CLI-2-288553).doc]

国务院关于印发“十三五”卫生与健康规划的通知

**发布部门：** [国务院](https://www.pkulaw.com/law/chl?Aggs.IssueDepartment=5)

**发文字号：**国发〔2016〕77号

**发布日期：**2016.12.27

**实施日期：**2016.12.27

**时效性：** [现行有效](https://www.pkulaw.com/law/chl?Aggs.TimelinessDic=01)

**效力级别：** [国务院规范性文件](https://www.pkulaw.com/law/chl?Aggs.EffectivenessDic=XC0203)

**法规类别：** [卫生综合规定](https://www.pkulaw.com/law/chl?Aggs.Category=09601)

国务院关于印发“十三五”卫生与健康规划的通知
（国发〔2016〕77号）

各省、自治区、直辖市人民政府，国务院各部委、各直属机构：
　　现将《“十三五”卫生与健康规划》印发给你们，请认真贯彻执行。

国务院
2016年12月27日

　　“十三五”卫生与健康规划

　　为推进健康中国建设，根据《[中华人民共和国国民经济和社会发展第十三个五年规划纲要](https://www.pkulaw.com/chl/3fd7b48d6cb8d951bdfb.html)》和《[“健康中国2030”规划纲要](https://www.pkulaw.com/chl/a55bb2910f6162efbdfb.html)》，编制本规划。
　　**一、**规划背景
　　（一）“十二五”时期取得的成就。
　　“十二五”时期，深化医药卫生体制改革加快实施，卫生与健康事业获得长足发展，人民健康水平持续提高。2015年人均预期寿命达到76.34岁，比2010年提高1.51岁，婴儿死亡率由13.1‰下降到8.1‰，5岁以下儿童死亡率由16.4‰下降到10.7‰，孕产妇死亡率由30/10万下降到20.1/10万，居民主要健康指标总体上优于中高收入国家平均水平，人口年均自然增长率为4.97‰，“十二五”卫生与健康事业有关规划确定的主要目标和任务如期完成。
　　医药卫生体制改革深入推进，取得重大进展和明显成效。全民医保体系加快健全，基本医保参保率保持在95%以上，城乡居民大病保险、重特大疾病医疗救助、疾病应急救助全面推开。公立医院改革稳步推进，县级公立医院综合改革全面实施，城市公立医院综合改革试点持续拓展深化，以省为单位实施综合医改试点取得积极进展。国家基本药物制度得到巩固完善，基层医疗卫生机构综合改革持续深化。社会办医加快发展。个人卫生支出占卫生总费用的比重由35.29%下降到29.27%。
　　医疗卫生服务体系不断完善，服务能力大幅提升。2015年，每千人口医疗卫生机构床位数增加到5.11张，执业（助理）医师数增加到2.22人，注册护士数增加到2.37人。医疗卫生机构基础设施条件持续改善。住院医师规范化培训制度初步建立，以全科医生为重点的基层医疗卫生人才队伍建设加快推进，2015年，每万人口全科医生数达到1.38人。有序推进分级诊疗制度建设，广泛开展“进一步改善医疗服务行动计划”等活动，初步建立预防化解医疗纠纷的长效机制。全面加强人口健康信息化建设。
　　生育政策逐步调整完善，计划生育服务管理改革统筹推进。平稳实施单独两孩政策。截至2015年底，近200万对单独夫妇提出再生育申请。研究启动全面两孩政策。妇幼保健和计划生育机构改革有序开展，计划生育服务管理改革扎实推进。出生人口性别比连续7年下降。国家免费孕前优生健康检查项目扩大到全国所有县（市、区），出生缺陷综合防治不断推进。流动人口免费计划生育服务覆盖率达到89.2%。
　　基本公共卫生服务均等化水平稳步提高，重大疾病防治成效显著。基本公共卫生服务人均经费补助标准提高到40元，服务内容增加到12类45项。艾滋病疫情控制在低流行水平，肺结核报告发病率下降到63.4/10万，所有血吸虫病流行县达到传播控制标准，基本消除或控制重点地方病危害。初步建立起慢性病防治体系，严重精神障碍防治网络不断完善。爱国卫生运动深入开展。居民健康素养水平稳步提升。推广血液筛查核酸检测，血液安全水平进一步提高。联防联控工作机制不断完善，成功防范和应对人感染禽流感等突发急性传染病和公共卫生事件。卫生计生综合监督执法进一步加强。食品安全标准与监测评估工作扎实推进。
　　中医药服务能力不断提升，中医药事业得到较快发展。多层次、广覆盖的中医药服务网络基本建立。基层中医药服务能力明显提升，全国超过95%的社区卫生服务中心、90%的乡镇卫生院、80%的社区卫生服务站、60%的村卫生室能够提供中医药服务。推动中医药科技进步，不断拓展中医药健康服务新业态。中医药“走出去”迈出重要步伐。
　　城乡居民健康差异进一步缩小，医疗卫生服务可及性、服务质量、服务效率和群众满意度显著提高，卫生与健康事业国际影响力凸显，为稳增长、促改革、调结构、惠民生作出了重要贡献，为全面建成小康社会、实现人人享有基本医疗卫生服务打下了坚实的基础。
　　（二）“十三五”时期面临的机遇和挑战。
　　党中央、国务院高度重视卫生与健康事业发展，提出推进健康中国建设，将卫生与健康事业发展摆在了经济社会发展全局的重要位置。人民群众对全面建成小康社会美好生活的追求激发多层次、多样化的健康需求，为健康服务业创造更为广阔的发展空间。全面依法治国深入推进，为提升卫生与健康治理体系和治理能力现代化水平提供坚实的法治保障。卫生与健康事业发展面临难得的历史机遇。
　　同时，卫生与健康事业发展也面临新的挑战。人口结构性问题日益突出，出生人口素质有待提高。全面两孩政策实施，老龄化进程加速，城镇化率不断提高，部分地区医疗卫生资源供需矛盾将更加突出。经济社会转型中居民生活环境与生活方式快速变化，慢性病成为主要的健康问题。重大传染病和重点寄生虫病等疾病威胁持续存在。境内外交流的日趋频繁加大传染病疫情和病媒生物输入风险。大气等环境污染和食品安全问题严重影响人民健康。经济发展进入新常态，互联网等新兴信息技术快速发展，要求卫生与健康领域加快转变发展方式，创新服务模式和管理方式。
　　此外，制约卫生与健康事业改革发展的内部结构性问题依然存在。一是资源总量不足、布局结构不合理尚未根本改变，优质医疗资源尤其缺乏。二是基层服务能力仍是突出的薄弱环节，基层医务人员技术水平亟待提高，服务设施和条件需要持续改善。三是深化改革需要进一步破解深层次的体制机制矛盾。四是计划生育工作思路和方法亟需转变。

　　**二、**指导思想和发展目标
　　（一）指导思想。高举中国特色社会主义伟大旗帜，全面贯彻党的十八大和十八届三中、四中、五中、六中全会精神，以马克思列宁主义、毛泽东思想、邓小平理论、“三个代表”重要思想、科学发展观为指导，深入贯彻习近平总书记系列重要讲话精神，紧紧围绕统筹推进“五位一体”总体布局和协调推进“四个全面”战略布局，认真落实党中央、国务院决策部署，牢固树立和贯彻落实创新、协调、绿色、开放、共享的发展理念，坚持以人民为中心的发展思想，坚持正确的卫生与健康工作方针，坚持计划生育基本国策，把人民健康放在优先发展的战略地位，以改革创新为动力，以促健康、转模式、强基层、重保障为着力点，更加注重预防为主和健康促进，更加注重工作重心下移和资源下沉，更加注重提高服务质量和水平，实现发展方式由以治病为中心向以健康为中心转变，显著提高人民健康水平，奋力推进健康中国建设。
　　（二）发展目标。到2020年，覆盖城乡居民的基本医疗卫生制度基本建立，实现人人享有基本医疗卫生服务，人均预期寿命在2015年基础上提高1岁。
　　--制度体系更加成熟定型。卫生计生法律制度进一步健全，治理体系和治理能力现代化水平不断提升，健康融入所有政策取得积极进展。
　　--健康服务体系持续完善。医疗卫生服务能力大幅提升，更好满足人民群众基本医疗卫生服务需求和多样化、多层次健康需求。
　　--疾病预防控制成效显著。预防为主，关口前移，普及健康生活方式，提升居民健康素养，有效控制健康危险因素，消除一批重大疾病。
　　--健康服务模式实现转变。机构间的分工协作更加紧密，家庭医生签约服务制度基本全覆盖，符合国情的分级诊疗制度基本建立。
　　--适度生育水平得到保持。全面两孩政策平稳实施，计划生育服务管理制度较为完善。
　　主　要　发　展　指　标

| 领域 | 主要指标 | 单位 | 2020年 | 2015年 | 指标性质 |
| --- | --- | --- | --- | --- | --- |
| 健康水平 | 人均预期寿命 | 岁 | ＞77.3 | 76.34 | 预期性 |
|  | 孕产妇死亡率 | /10万 | ＜18 | 20.1 | 预期性 |
|  | 婴儿死亡率 | ‰ | ＜7.5 | 8.1 | 预期性 |
|  | 5岁以下儿童死亡率 | ‰ | ＜9.5 | 10.7 | 预期性 |
| 疾病防控 | 居民健康素养水平 | % | ＞20 | 10 | 预期性 |
|  | 以乡（镇、街道）为单位适龄儿童免疫规划疫苗接种率 | % | ＞90 | ＞90 | 约束性 |
|  | 肺结核发病率 | /10万 | ＜58 | 63.4 | 预期性 |
|  | 因心脑血管疾病、癌症、慢性呼吸系统疾病和糖尿病导致的过早死亡率 | % | 比2015年降低10% | 18.5 | 预期性 |
| 妇幼健康 | 孕产妇系统管理率 | % | ＞90 | ＞90 | 约束性 |
|  | 3岁以下儿童系统管理率 | % | ＞90 | ＞90 | 约束性 |
|  | 孕前优生健康检查目标人群覆盖率 | % | ＞80 | ＞80 | 预期性 |
| 医疗服务 | 三级医院平均住院日 | 天 | ＜8 | 10.2 | 预期性 |
|  | 院内感染发生率 | % | ＜3.2 | 3.5 | 预期性 |
|  | 30天再住院率 | % | ＜2.4 | 2.65 | 预期性 |
|  | 门诊处方抗菌药物使用率 | % | ＜10 | ＜11 | 预期性 |
| 计划生育 | 总人口 | 亿人 | 14.2左右 | 13.7 | 预期性 |
|  | 总和生育率 |  | 1.8左右 | 1.5-1.6 | 预期性 |
|  | 出生人口性别比 |  | ＜112 | 113.5 | 约束性 |
| 医疗卫生服务体系 | 每千人口医疗卫生机构床位数 | 张 | ＜6 | 5.11 | 预期性 |
|  | 每千人口执业（助理）医师数 | 人 | ＞2.5 | 2.22 | 预期性 |
|  | 每千人口注册护士数 | 人 | ＞3.14 | 2.37 | 预期性 |
|  | 每万人口全科医生数 | 人 | ＞2 | 1.38 | 约束性 |
|  | 社会办医院床位占医院床位总数的比重 | % | ＞30 | 19.4 | 预期性 |
| 医疗卫生保障政策 | 范围内住院费用基本医保支付比例 | % | 75左右 | 75左右 | 预期性 |
|  | 个人卫生支出占卫生总费用的比重 | % | 28左右 | 29.27 | 约束性 |

　　**三、**主要任务
　　（一）加强重大疾病防治。
　　推进防治结合。建立专业公共卫生机构、综合性医院和专科医院、基层医疗卫生机构“三位一体”的重大疾病防控机制，信息共享、互联互通，推进慢性病和精神疾病防、治、管整体融合发展。落实医疗卫生机构承担公共卫生任务的补偿政策，完善政府购买公共卫生服务机制。（国家卫生计生委、财政部负责）
　　实施慢性病综合防控。完善政府主导的慢性病综合防控协调机制，优化防控策略，建立以基层为重点的慢性病防控体系，加强国家综合防控示范区建设，覆盖全国15%以上的县（市、区）。加强脑卒中等慢性病的筛查和早期发现，针对高发地区重点癌种开展早诊早治工作，早诊率达到55%，提高5年生存率。全面实施35岁以上人群首诊测血压，逐步开展血压血糖升高、血脂异常、超重肥胖等慢性病高危人群的患病风险评估和干预指导，将口腔健康检查和肺功能检测纳入常规体检。高血压和糖尿病患者健康管理人数分别达到1亿人和3500万人。健全死因监测、肿瘤登记报告和慢性病与营养监测制度。加强伤害预防和干预。（国家卫生计生委负责）
　　加强重大传染病防治。加强传染病监测预警、预防控制能力建设，法定传染病报告率达到95%以上，及时做好疫情调查处置。降低全人群乙肝病毒感染率。加强艾滋病检测、干预和随访，最大限度发现感染者和病人，为所有符合条件且愿意接受治疗的感染者和病人提供抗病毒治疗，将疫情控制在低流行水平。开展肺结核综合防治服务试点，加大一般就诊者肺结核发现力度，强化重点人群主动筛查，加强耐多药肺结核筛查和监测，规范患者全程治疗管理。有效应对霍乱、流感、手足口病、麻疹等重点传染病疫情。实施以传染源控制为主的狂犬病、布病、禽流感等人畜共患病综合治理策略。消除麻风病危害。建立已控制严重传染病防控能力储备机制。（国家卫生计生委牵头，农业部等相关部门参与）加强口岸卫生检疫能力建设，加强境外传染病监测预警和应急处置，推动口岸疑似传染病旅客接受免费传染病检测，严防外来重大传染病传入。（质检总局负责）
　　强化精神疾病防治。加强严重精神障碍患者报告登记、服务管理和救治救助，在册的严重精神障碍患者管理率达到80%以上。逐步建立和完善精神障碍患者社区康复服务体系。开展焦虑、抑郁等常见精神障碍早期筛查和干预试点，抑郁症治疗率显著提高。加强心理健康服务。（国家卫生计生委牵头，公安部、民政部、中国残联等相关部门和单位参与）
　　实施扩大国家免疫规划。夯实常规免疫，做好补充免疫和查漏补种，推进接种门诊规范化建设，提升预防接种管理质量。在全国范围内开展脊灰灭活疫苗替代工作，继续维持无脊灰状态。根据防病工作需要，适时调整国家免疫规划疫苗种类，逐步将安全有效、财政可负担的疫苗纳入国家免疫规划。加强疫苗可预防传染病监测。探索建立预防接种异常反应补偿保险机制。改革完善第二类疫苗集中采购机制，加强疫苗冷链管理，推进疫苗全程追溯体系建设，严禁销售非法疫苗。（国家卫生计生委牵头，财政部、食品药品监管总局、质检总局等相关部门参与）
　　做好重点寄生虫病及地方病防控工作。坚持以传染源控制为主的血吸虫病综合防治策略。加强登革热、疟疾等蚊媒传染病控制，全国实现消除疟疾目标。实施包虫病综合防治策略，基本控制包虫病流行。持续消除碘缺乏危害，人群碘营养总体处于适宜水平。保持基本消除大骨节病、克山病和燃煤污染型氟、砷中毒危害，有效控制饮水型地方性氟、砷中毒危害和饮茶型地氟病危害。（国家卫生计生委牵头，水利部、农业部等相关部门参与）
　　推进职业病防治工作。开展职业病危害普查和防控，加强尘肺病等重点职业病监测和职业健康风险评估。提高医用辐射防护监测与危害控制水平。提升医疗卫生机构职业病报告、职业健康检查和职业病诊断、鉴定、救治能力。加强职业人群健康教育，推动用人单位落实职业病防治主体责任，开展用人单位职业健康促进试点。（国家卫生计生委、安全监管总局负责）
　　加强突发事件卫生应急。加强突发公共卫生事件尤其是突发急性传染病综合监测、快速检测、风险评估和及时预警能力建设，提升突发事件卫生应急监测预警水平、应对能力和指挥效力，突发公共卫生事件预警信息响应率达到95%以上。加强卫生应急队伍建设，提高各级医疗卫生机构卫生应急准备和处置能力，鼠疫、人禽流感等突发急性传染病现场规范处置率达95%以上。完善重大自然灾害医学救援、突发公共卫生事件军地联防联控机制。建立并完善国家生物安全协调机制，倡导卫生应急社会参与。（国家卫生计生委、中央军委后勤保障部卫生局负责）

专栏1　重大疾病防治项目

慢性病综合防控：慢性病综合防控示范区，慢性病与营养监测及综合干预，癌症早诊早治，脑卒中、心血管病、慢性呼吸系统疾病筛查干预，高血压、糖尿病高危人群健康干预，重点人群口腔疾病综合干预。（国家卫生计生委负责）　　重大传染病防控：艾滋病防控，结核病防控，流感和不明原因肺炎监测，手足口病、狂犬病、布病、流行性出血热、登革热、麻风病等传染病的监测及早期干预，突发急性传染病防控。（国家卫生计生委负责）

精神疾病防治：严重精神障碍患者管理治疗，心理健康服务，精神卫生综合管理试点。（国家卫生计生委负责）

扩大国家免疫规划：扩大国家免疫规划，急性弛缓性麻痹病例及麻疹、乙肝等疫苗可预防重点传染病监测。（国家卫生计生委负责）

重点寄生虫病及地方病防控：血吸虫病防控，疟疾、包虫病等重点寄生虫病防治，重点地方病防控。（国家卫生计生委负责）

职业病防治：重点职业病监测与职业健康风险评估，职业性放射性疾病监测与职业健康风险评估，医疗卫生机构医用辐射防护监测。（国家卫生计生委负责）

基本公共卫生服务项目：居民健康档案、健康教育、预防接种、儿童健康管理、孕产妇健康管理、老年人健康管理、慢性病（高血压、Ⅱ型糖尿病）患者健康管理、严重精神障碍患者管理、结核病患者健康管理，中医药健康管理、卫生计生监督协管、传染病和突发公共卫生事件报告和处理等。（国家卫生计生委、国家中医药局、财政部负责）

　　（二）推动爱国卫生运动与健康促进。
　　着力改善城乡环境卫生面貌。深入推进卫生城镇创建，国家卫生城市比例提高到40%，国家卫生县城（乡镇）比例提高到5%。开展城乡环境卫生整洁行动，以城市环境卫生薄弱地段和农村垃圾污水处理、改厕为重点，完善城乡环境卫生基础设施和长效管理机制，加快推进农村生活污水治理和无害化卫生厕所建设，农村卫生厕所普及率达到85%以上，实施农村生活垃圾治理专项行动。加快实施农村饮水安全巩固提升工程，推动城镇供水设施向农村延伸，农村集中式供水卫生安全巡查覆盖90%以上的乡镇。科学防制病媒生物。推进多污染物综合防治和环境治理。加强大气污染综合治理，改善大气环境质量。推进重点流域水污染防治和土壤污染治理与修复。加强环境与健康综合监测和风险评估。（国家卫生计生委、环境保护部、住房城乡建设部、水利部负责）
　　全面推进健康城市和健康村镇建设。开展健康城市综合示范建设，形成可推广的健康城市建设模式。广泛开展健康社区、健康单位、健康学校、健康家庭建设，创新社会动员和群众参与工作方式，鼓励社会组织开展志愿服务、健康自我管理小组、社区健康讲堂等活动。开展健康城市建设效果评价，实现科学、动态管理。推进健康村镇建设，提高农村居民卫生素质和健康水平。健康城市和健康村镇工作体系基本健全，健康管理工作模式基本建立，建成一批健康城市建设示范市和健康村镇建设示范村镇。（国家卫生计生委负责）
　　深入开展全民健康教育和健康促进活动。广泛开展全民健康素养促进行动和健康中国行等活动，普及合理营养、合理用药、科学就医和灾害自救互救等知识，提高全民健康素养。加强健康科普规范化管理，建立健全健康知识和技能核心信息发布制度。倡导健康文明的生活方式，实施国民营养计划，引导群众加强自我健康管理，深入推进以减盐、减油、减糖、健康口腔、健康体重、健康骨骼为重点的全民健康生活方式行动，广泛宣传合理膳食、适量运动、戒烟限酒、心理平衡等健康科普知识，开展家庭和高危个体健康生活方式强化指导和干预。加强健康教育能力建设，推进医疗机构开展健康教育和健康促进工作。全面推进控烟履约工作，加快控烟立法，大力开展无烟环境建设，全面推进公共场所禁烟，强化戒烟服务，预防和控制被动吸烟。健全健康素养和烟草流行监测体系，15岁以上人群烟草使用流行率控制在25%以下。（国家卫生计生委牵头，中央宣传部、工业和信息化部、体育总局、国务院法制办等相关部门参与）
　　增强人民体质。推进基本公共体育服务体系建设，统筹建设全民健身场地设施，构建场地设施网络和城市社区15分钟健身圈，人均体育场地面积达到1.8平方米。推动公共体育设施免费或低收费开放，逐步对社会开放学校体育场馆等运动健身场所。广泛组织开展全民健身运动，大力发展群众健身休闲项目，鼓励实行工间健身制度，切实保证中小学生每天一小时校园体育活动。加强全民健身组织建设和人才培养。开展国民体质监测和全民健身活动状况调查，为群众提供个性化的科学健身指导服务，经常参加体育锻炼的人数达到4.35亿人。（体育总局、教育部负责）

专栏2　爱国卫生与健康促进项目

健康城市和健康村镇：健康城市与健康村镇综合试点，农村改厕，病媒生物监测，城乡环境卫生整治示范村建设。（国家卫生计生委负责）

环境健康危害因素监测：城乡饮用水卫生监测，农村环境卫生监测，公共场所健康危害因素监测，空气污染等对人群健康影响监测，人体生物监测。（国家卫生计生委负责）

全民健康生活方式：减少烟草危害行动，推广减盐、减油、减糖、健康体重、健康口腔、健康骨骼等专项行动。（国家卫生计生委负责）

健康教育：健康素养促进行动，健康中国行活动，健康家庭行动。（国家卫生计生委负责）

全民健身：全民健身场地设施建设，运动促进健康专项行动，青少年体育活动促进计划。（体育总局负责）

　　（三）加强妇幼卫生保健和生育服务。
　　保障妇幼健康。向孕产妇提供生育全过程的基本医疗保健服务，进一步提高孕产妇、新生儿危急重症救治能力，有效降低孕产妇死亡率和婴儿死亡率。加强高危孕产妇专案管理，预防艾滋病、梅毒、乙肝母婴传播，保障母婴安全。大力倡导婚检，继续实施免费孕前优生健康检查，落实出生缺陷三级预防措施，建立覆盖城乡，涵盖孕前、孕期、新生儿各阶段的出生缺陷防治服务制度，有效减少出生缺陷的发生。加大妇女常见病防治力度，妇女常见病定期筛查率达到80%以上，逐步扩大妇女“两癌”检查项目覆盖范围，提高宫颈癌和乳腺癌的早诊早治率。加强儿童疾病防治和意外伤害预防。大力推行母乳喂养，开展婴幼儿营养与喂养、生长发育及心理行为指导，扩大贫困地区儿童营养改善和新生儿疾病筛查项目覆盖范围，5岁以下儿童生长迟缓率控制在7%以下，低体重率降低到5%以下。加强计划生育技术服务，落实国家规定的免费计划生育技术服务基本项目，全面推行知情选择，普及避孕节育、优生优育和生殖健康知识，提高药具服务的可及性和便捷性，做好再生育技术服务指导，提高生殖健康水平。（国家卫生计生委、财政部负责）
　　关爱青少年健康。以中小学为重点，加强学校卫生工作。开展学生健康危害因素监测与评价，加强学生近视、龋齿、肥胖等常见病防治工作。加大学校健康教育与健康促进工作力度，将健康教育纳入国民教育体系。在总结好国家试点经验的基础上，实施农村义务教育学生营养改善计划，建立学生营养与健康监测评估制度，加大对学校集体供餐的食品安全和营养质量监管、指导力度。加强学校结核病、艾滋病等传染病防治和心理健康服务。关爱青少年生殖健康，减少非意愿妊娠。加强托幼机构卫生保健工作，托幼机构卫生保健指导实现全覆盖。（国家卫生计生委、教育部、食品药品监管总局负责）
　　（四）发展老年健康服务。
　　提高老年人健康素养。开展老年常见病、慢性病的健康指导和综合干预，推广以慢病管理、中医药和老年营养运动干预为主的适宜技术，65岁以上老年人健康管理率达到70%以上，有效改善老年人群营养健康状况，降低失能风险。开展长期护理保险试点，探索建立长期护理保险制度。开展老年心理健康和心理关怀服务。积极防治老年痴呆症。（国家卫生计生委、人力资源社会保障部、保监会负责）
　　健全老年健康服务体系。重点发展社区健康养老服务，提高基层医疗卫生机构为居家老年人提供上门服务的能力。所有医疗机构开设为老年人提供挂号、就医等便利服务的绿色通道，加强综合性医院老年病科建设。提高基层医疗卫生机构康复、护理床位占比，鼓励其根据服务需求增设老年养护、安宁疗护病床。完善治疗-康复-长期护理服务链，发展和加强康复、老年病、长期护理、慢性病管理、安宁疗护等接续性医疗机构。（国家卫生计生委负责）
　　推动医疗卫生与养老服务融合发展。统筹医疗卫生与养老服务资源，创新健康养老服务模式，建立健全医疗机构与养老机构之间的业务协作机制。鼓励二级以上综合性医院与养老机构开展对口支援、合作共建。推动二级以上综合性医院与老年护理院、康复疗养机构、养老机构内设医疗机构等之间的转诊与合作。支持养老机构按规定开办医疗机构，开展老年病、康复、护理、中医和安宁疗护等服务。推动中医药与养老结合，充分发挥中医药在养生保健和疾病康复领域优势。（国家卫生计生委、民政部牵头，国家中医药局参与）
　　（五）促进贫困人口等重点人群健康。
　　实施健康扶贫工程。保障贫困人口享有基本医疗卫生服务，努力防止因病致贫、因病返贫。对符合条件的贫困人口参加城乡居民基本医疗保险个人缴费部分按规定由财政给予补贴。新型农村合作医疗和大病保险制度对贫困人口实行政策倾斜，门诊统筹率先覆盖所有贫困地区。将贫困人口按规定纳入重特大疾病医疗救助范围。对患大病和慢性病的农村贫困人口进行分类救治。建立贫困人口健康卡。明显改善贫困地区医疗服务能力。实施军地三级医院与集中连片特困地区县和国家扶贫开发工作重点县县级医院稳定持续的一对一帮扶，深入推进二级以上医疗机构对口帮扶贫困县乡镇卫生院。积极促进远程医疗服务向贫困地区延伸。（国家卫生计生委牵头，国务院扶贫办、民政部、人力资源社会保障部、财政部、中央军委后勤保障部卫生局、保监会、国家中医药局等相关部门参与）
　　维护流动人口健康。按照常住人口（或服务人口）配置资源，将流动人口纳入流入地卫生计生服务体系。全面推进流动人口基本公共卫生计生服务均等化，流动人口目标人群基本公共卫生计生服务覆盖率达到90%。完善基本医保关系转移接续办法，提高流动人口医疗保障水平。做好流动人口聚居地突发公共卫生事件应对。广泛开展流动人口健康促进行动，提高流动人口健康素养水平。深化流动人口全国“一盘棋”机制建设。关怀关爱留守人群特别是留守儿童，在40个县开展留守儿童健康教育项目，促进社会融合。（国家卫生计生委、人力资源社会保障部、民政部负责）
　　确保残疾人享有健康服务。城乡残疾人普遍享有基本医疗保障，加大符合条件的低收入残疾人医疗救助力度，逐步将符合条件的残疾人医疗康复项目按规定纳入基本医疗保险支付范围。完善医疗卫生机构无障碍设施。实施精准康复服务行动，以残疾儿童和持证残疾人为重点，有康复需求的残疾人接受基本康复服务的比例达到80%。加强残疾人健康管理和社区康复。（中国残联、国家卫生计生委、人力资源社会保障部、民政部等相关部门和单位负责）

专栏3　重点人群健康改善项目

健康老龄化：老年人健康管理，老年心理健康与心理关怀，医养结合试点示范，长期护理保险试点。（国家卫生计生委、人力资源社会保障部、民政部负责）

健康妇幼：农村妇女“两癌”检查，计划生育技术服务基本项目和避孕药具，再生育技术服务，预防艾滋病、梅毒、乙肝母婴传播。（国家卫生计生委、财政部负责）

出生缺陷综合防治：农村夫妇免费孕前优生健康检查、增补叶酸预防神经管缺陷、孕期唐氏综合症产前筛查和产前诊断、新生儿疾病筛查、地中海贫血防控、先天性心脏病防治。（国家卫生计生委、财政部负责）

青少年健康：学生健康危害因素和常见病监测及防治，心理健康教育。（国家卫生计生委、教育部负责）

健康扶贫：对符合条件的因病致贫人口提供医疗救助，省级巡回医疗队建设，三级医院与重点贫困县医院对口帮扶，二级以上医疗卫生机构对口帮扶贫困县卫生院。（国家卫生计生委、国务院扶贫办、民政部负责）

流动人口健康维护：流动人口基本公共卫生计生服务均等化、流动人口健康促进行动、流动人口卫生计生动态监测。（国家卫生计生委负责）

　　（六）完善计划生育政策。
　　实施全面两孩政策。合理配置妇幼保健、儿童照料、学前和中小学教育、社会保障等资源，满足新增公共服务需求。加强分类指导，鼓励按政策生育。做好政策调整前后计划生育政策和相关经济社会政策的衔接，维护群众的合法权益。加强政策解读和宣传倡导，依法依规查处政策外多孩生育，维护良好生育秩序。完善出生人口信息管理，加强出生人口监测预警，及时把握出生人口动态。（国家卫生计生委牵头，国家发展改革委、教育部、人力资源社会保障部等相关部门参与）
　　改革完善计划生育服务管理。统筹推进生育政策、服务管理制度、家庭发展支持体系和治理机制综合改革，推动人口和计划生育工作由控制人口数量为主向调控总量、提升素质和优化结构并举转变，由管理为主向更加注重服务家庭转变，由主要依靠政府力量向政府、社会和公民多元共治转变，更加注重宣传倡导、服务关怀、政策引导和依法行政。深入开展计划生育优质服务先进单位创建活动。加强计划生育服务管理能力建设，稳定基层工作网络和队伍。实行生育登记服务制度。全面推行网上办事和承诺制。充分发挥计划生育协会等群团组织和其他社会组织的作用，深化诚信计生和基层群众自治活动。（国家卫生计生委负责）
　　提高计划生育家庭发展能力。完善计划生育家庭奖励和扶助政策体系，加大对计划生育家庭的扶助力度，加强对计划生育特殊家庭的扶助和关爱。继续实施农村部分计划生育家庭奖励扶助制度和计划生育家庭特别扶助制度，实行扶助标准动态调整。在生育水平较高、生态环境脆弱、扶贫任务艰巨的西部地区，着力做好计划生育家庭奖励扶助等工作。坚持男女平等，严厉打击非医学需要的胎儿性别鉴定和选择性别人工终止妊娠行为，综合治理出生人口性别比偏高问题。深入开展关爱女孩行动，做好符合条件的计划生育女孩及女孩家庭扶助工作，提升计划生育女孩家庭发展能力。（国家卫生计生委、财政部负责）
　　坚持和完善计划生育目标管理责任制。坚持计划生育党政一把手亲自抓、负总责，坚持计划生育兼职委员和领导小组制度，强化各地区各部门齐抓共管的工作格局。建立健全与新时期形势任务相适应、科学合理、便捷高效的计划生育目标管理责任制考核体系和运行机制，落实“一票否决”。（国家卫生计生委负责）

专栏4　计划生育服务管理项目

计划生育服务管理：调整完善生育政策监测，基层计划生育服务管理能力建设，出生人口性别结构平衡促进，社会性别平等促进，农村部分计划生育家庭奖励扶助，计划生育家庭特别扶助，家庭发展追踪调查，创建幸福家庭活动。（国家卫生计生委、财政部负责）

　　（七）提升医疗服务水平。
　　实行分级诊疗。以提高基层医疗服务能力为重点，以常见病、多发病、慢性病分级诊疗为突破口，形成科学合理的就医秩序，基本实现基层首诊、双向转诊、急慢分治、上下联动。明确各级各类医疗机构诊疗服务功能定位，控制三级医院普通门诊规模，支持和引导病人优先到基层医疗卫生机构就诊，由基层医疗卫生机构逐步承担公立医院的普通门诊、稳定期和恢复期康复以及慢性病护理等服务。鼓励二级以上医院成立全科医学科。推进全科医生（家庭医生）能力提高及电子健康档案等工作，发挥全科医生（家庭医生）的居民健康“守门人”作用，实施家庭医生签约服务制度，优先覆盖老年人、孕产妇、儿童、残疾人等人群，以及高血压、糖尿病、结核病等慢性疾病和严重精神障碍患者等。推进和规范医师多点执业。完善不同级别医疗机构的医保差异化支付和价格政策，促进各级各类医疗卫生机构分工协作机制的建立。将军队医疗机构全面纳入分级诊疗体系。（国家卫生计生委牵头，国家发展改革委、人力资源社会保障部、中央军委后勤保障部卫生局等相关部门参与）
　　提高医疗质量安全水平。规范诊疗行为，全面实施临床路径，加强重大疾病规范化诊疗管理，保障医疗安全。加强药师队伍建设，实施遏制细菌耐药国家行动计划，以抗菌药物为重点推进合理用药，加强处方监管，提高临床用药的安全性、有效性。加强医疗质量监管，健全医疗技术临床应用管理制度。逐步完善国家、省级、地市级医疗质量控制网络。建立科学的医疗绩效评价机制以及医疗质量控制动态监测和反馈机制，健全医疗安全保障体系，实现医疗质量和医疗安全水平持续提升。持续提高护理技术水平，充分发挥护理在提升医疗质量中的积极作用。加强医师执业管理，健全医师定期考核制度。完善医疗机构登记和医师注册制度，采用电子证照等信息化手段，实现医疗执业活动动态、全过程管理。建立以控制不合理费用为重点的内审制度，规范医务人员医疗卫生服务行为。（国家卫生计生委、中央军委后勤保障部卫生局负责）
　　加强临床服务能力建设。加强对临床专科建设发展的规划引导和支持，提升临床专科整体服务能力与水平。加强临床重点专科建设，以发展优质医疗资源为目标，建设一批高水平临床专科，重点支持肿瘤、心脑血管、儿科、精神、感染、妇产等薄弱领域重点专科诊疗能力提升，发挥其示范、引领、带动和辐射作用，促进医疗服务体系协调发展。针对各省专科现状和发展需求加强薄弱专科能力建设，增加优质医疗资源总量，提升专科综合服务能力，降低省外就医率。加强县域内常见病、多发病相关专业，传染病、精神疾病及急诊急救、重症医学、血液透析、妇产科、儿科、中医等临床专科建设，全面提升县级公立医院综合能力，将县域内就诊率提高到90%左右，基本实现大病不出县。加强基层医疗卫生机构服务能力建设，提高常见病、多发病和慢性病的诊治、康复服务能力。进一步拓展中心乡镇卫生院的功能，提升急诊抢救、二级以下常规手术、正常分娩、高危孕产妇筛查、儿科等医疗服务能力。继续开展防盲治盲和防聋治聋工作。（国家卫生计生委、科技部负责）
　　改善医疗服务。优化诊区设施布局，营造温馨就诊环境。推进预约诊疗服务，有效分流就诊患者。合理调配诊疗资源，推行日间手术，加强急诊力量，畅通急诊绿色通道。发挥信息技术优势，推行电子病历，提供诊疗信息、费用结算、信息查询等服务，完善入院、出院、转院服务流程，改善患者就医体验。全面实施优质护理服务。大力推进医疗联合体内医疗机构检查、检验结果互认和同城同级医疗机构检查、检验结果互认工作。强化患者安全管理。推进社区卫生服务提升工程和建设群众满意乡镇卫生院活动。保持打击涉医违法犯罪行为的高压态势，健全院内调解、人民调解、司法调解、医疗风险分担机制有机结合的“三调解一保险”制度体系，妥善化解医疗纠纷，构建和谐医患关系。（国家卫生计生委、公安部、保监会负责）
　　完善血液供应保障机制。继续提高人口献血率，无偿献血人次数和献血量增长水平与当地医疗服务需求增长水平相适应。开展血液安全风险监测，巩固血液核酸检测全覆盖成果，健全血液质量控制和改进体系，推进临床合理用血。（国家卫生计生委负责）

专栏5　医疗服务改进项目

基层医疗卫生服务：电子健康档案，健康卡。（国家卫生计生委负责）

分级诊疗：慢性病一体化诊疗服务试点，家庭医生签约服务。（国家卫生计生委负责）

医疗服务能力：临床专科能力建设。（国家卫生计生委、财政部负责）

医疗质量安全管理：医疗质量管理与控制体系建设，医院感染管理监测和质量持续改进，血液安全。（国家卫生计生委负责）

　　（八）推动中医药传承创新发展。
　　加强中医药传承创新。加快发展中医医疗服务，健全覆盖城乡的中医医疗服务体系，加强中医重点专科建设，创新中医医院服务模式。充分利用中医药技术方法和现代科学技术，提高危急重症、疑难复杂疾病的中医诊疗服务能力和中医优势病种的中医门诊诊疗服务能力。大力发展中医养生保健服务，推广中医养生保健技术与方法，促进中医养生保健机构规范发展。加强中医临床研究基地和科研机构建设，强化中医理论基础研究，推进中医药标准化、现代化。加强中医药传统知识保护，编撰出版《中华医藏》，建立中医药传统知识保护数据库。完善中医药人才培养体系，加快推进各层次各类型中医药人才培养，健全国医大师评选表彰制度，完善中医药人才评价机制。推进中医药文化传承和发展，弘扬中医药文化精髓，实施中医药健康文化素养提升工程。开展中药资源普查，加强中药资源保护利用，推进中药材规范化种植养殖，加强中药疗效与质量保障体系建设，健全中药材流通追溯机制，促进中药资源可持续发展，提升中药产业发展水平。积极发展民族医药事业。推广中医药适宜技术。（国家中医药局、国家卫生计生委、国家发展改革委、工业和信息化部、教育部、科技部、商务部、农业部负责）
　　推进中西医协调发展。健全中医药学与现代医学互为补充、惠及大众的中医药健康服务体系。加强中西医结合，促进中医药原创思维和现代快速发展的新技术、新方法有机结合，寻找防治疾病的创新路径和手段，促进中西医药协调发展。加强中西医临床协作，提高重大疑难病、急危重症临床疗效。加强高层次中西医结合人才培养，鼓励西医师全面、系统学习中医。中医类别医师可根据临床需要使用与专业相关的现代医药方法和技术，参加与自身专业相关的特殊准入医疗技术培训。支持非中医类别医师学习中医药理论知识和技能，并在临床实践中应用。实施基层中医药服务能力提升工程，提升基层西医和中医两种手段综合服务能力，力争使所有社区卫生服务机构、乡镇卫生院和70%的村卫生室具备与其功能相适应的中医药服务能力。（国家中医药局、国家卫生计生委、国家发展改革委负责）

专栏6　中医药传承与创新项目

中医药传承与创新：全面改善中医医院基础设施条件，支持中医馆建设。提升省级中医药机构科研能力。支持中医重点学科和重点专科（专病）建设。加强中医药人才培养。开展中药资源普查。实施中医药传承工程、中医临床优势培育工程、基层中医药服务能力提升工程。（国家中医药局、国家卫生计生委、国家发展改革委、教育部负责）

　　（九）强化综合监督执法与食品药品安全监管。
　　加强监督执法体系建设。改革和完善卫生计生综合监督行政执法工作，整合卫生计生执法资源，健全完善卫生计生监督执法体系，推动执法重心下移。完善常态化监管机制，加强事中事后监管，实行“双随机”抽查机制，加强全行业监管。建立健全国家重点监督抽检网络。强化依法行政，严格行政执法，提高卫生计生行政执法能力和水平。开展重要卫生计生法律法规落实情况监督检查。健全行政执法制度，围绕社会高度关注、涉及群众切身利益的卫生计生突出问题，大力开展专项整治、重点监督检查和经常性督导检查，严厉打击违法行为。建立健全监督执法责任制和责任追究制。加强卫生计生综合监督行政执法队伍建设。强化监督执法能力建设，完善监管信息系统，推进信息披露和公开，提高监督执法效率。建立健全行业诚信体系和失信联合惩戒机制，建立医药卫生行业“黑名单”制度。（国家卫生计生委负责）
　　强化食品药品安全监管。实施食品安全战略，完善食品安全法规制度。健全国家食品安全标准体系，完善标准管理制度，加快制定重金属、农药残留、兽药残留等重点食品安全标准，完成不少于300项标准的制定、修订。完善食品安全风险监测与评估工作网络，开展食品安全风险监测，推进食物消费量调查和总膳食研究，系统完成25项食品化学污染物等物质的风险评估。建立健全食品安全事故流行病学调查机制，食源性疾病监测报告网络覆盖县乡村。实施国家药品标准提高行动计划，开展仿制药质量和疗效一致性评价。健全药品医疗器械监管技术支撑体系，提高检验检测能力，提升对药品医疗器械不良反应事件的监测评价和风险预警水平。加强药物临床试验机构建设。健全严密高效、社会共治的食品药品安全治理体系。加大农村食品药品安全治理力度，完善对网络销售食品药品的监管。加强食品药品进口监管。（国家卫生计生委、食品药品监管总局、农业部、质检总局、中央军委后勤保障部卫生局负责）

专栏7　综合监督与食品安全项目

国家重点监督抽检网络建设：国家重点监督抽检，医疗机构医疗卫生和传染病防治监督抽检；公共场所、学校和供水单位公共卫生监督抽检；法律、法规落实情况监督检查；计划生育技术服务机构、采供血机构、放射卫生技术服务机构、消毒产品生产企业和涉水产品生产企业监督抽检。（国家卫生计生委负责）

食品安全标准与监测评估：食品安全标准体系建设，整合现有资源进行食品安全风险监测评估网络和食源性疾病监测报告网络与溯源平台建设，食源性疾病管理和食品安全事故流行病学调查能力建设。（国家卫生计生委负责）

　　（十）加快健康产业发展。
　　大力发展社会办医。鼓励社会力量兴办健康服务业，按照每千常住人口不低于1.5张床位为社会力量办医预留规划空间，同步预留诊疗科目设置和大型医用设备配置空间。个体诊所设置不受规划布局限制。优先支持举办非营利性医疗机构，推进非营利性民营医院和公立医院同等待遇。放宽社会力量举办医疗机构的服务领域要求，支持社会力量以多种形式参与健康服务。发展专业性医院管理集团，推动社会力量办医疗机构上水平发展。鼓励社会力量发展儿科、精神科、老年病、长期护理、口腔保健、康复、安宁疗护等资源稀缺及满足多元需求的服务。大力推动医师多点执业，鼓励医师到基层医疗卫生机构多点执业。大力发展第三方服务，引导发展专业的医学检验中心和影像中心等。公立医院资源丰富的地区，社会力量可以多种形式参与国有企业所办医疗机构等部分公立医院改制重组。鼓励公立医院与社会力量共同举办新的非营利性医疗机构，满足群众多层次医疗服务需求。强化行业监管和行业自律，规范市场秩序，保障医疗质量和安全。（国家卫生计生委、国家发展改革委、商务部、国务院国资委负责）
　　积极发展健康服务新业态。提高健康管理与促进服务水平。推动健康医疗旅游发展，开发有特色的中医药健康旅游产品，提升医疗服务的国际化水平。培育健康医疗大数据应用新业态。加强健康体检的规范化管理。发展中医药健康服务。打造一批知名品牌和良性循环的健康服务产业集群，并形成一定的国际竞争力。开拓发展国际旅行健康服务。（国家卫生计生委、质检总局、国家旅游局、国家中医药局负责）
　　加快发展商业健康保险。鼓励企业和个人通过参加商业保险及多种形式的补充保险解决基本医保之外的需求。鼓励商业保险机构积极开发与健康管理服务相关的健康保险产品，加强健康风险评估和干预。加快发展医疗责任保险、医疗意外保险，探索发展多种形式的医疗执业保险。（保监会负责）
　　创新发展药品、医疗器械等产业。鼓励创新药和临床急需品种上市。在加强行业规范的基础上，推动基因检测、细胞治疗等新技术的发展。引导企业提高创新质量，培育重大产品。支持企业兼并重组、强强联合，培育具有国际竞争力的大型企业，提高产业集中度。大力发展智能健康医疗装备。支持提升医疗设备的产业化能力和质量水平，推进发展应用。开发可穿戴生理信息监测设备、便携式诊断设备等移动医疗产品和可实现远程监护、诊断、治疗指导的远程医疗系统。（工业和信息化部、国家卫生计生委、食品药品监管总局、科技部、国家发展改革委负责）

专栏8　健康产业发展项目

健康服务业发展：社会办医示范机构、健康管理与促进服务示范机构、健康医疗旅游示范基地建设。（国家卫生计生委、国家发展改革委、财政部、国家旅游局负责）

　　（十一）加强卫生计生服务体系建设。
　　优化医疗卫生服务体系。统筹规划区域卫生资源，按照军民融合发展战略将军队医院纳入驻地有关规划，优化医疗卫生机构布局，推动京津冀医疗卫生协同发展，促进医疗资源向中西部地区倾斜、向基层和农村流动，缩小区域之间基本医疗卫生服务的差距。强基层、补短板，提高妇幼健康、公共卫生、肿瘤、精神、产科、儿科、康复、护理等急需领域医疗服务能力。构建整合型医疗卫生服务体系，提高资源使用效率，避免重复建设。（国家卫生计生委、中央军委后勤保障部卫生局负责）
　　推动公立医院科学发展。对新建城区、郊区、卫星城区等薄弱区域，政府要有计划、有步骤建设公立医疗卫生机构，满足群众基本医疗卫生需求。控制公立医院规模过快扩张。依托现有资源，合理规划与设置国家医学中心及国家、省级区域医疗中心，继续加强县级公立医院建设，改善县级医院业务用房和装备条件，提高服务能力。加强大型医用设备配置规划和准入管理，严控公立医院超常装备，逐步建立大型设备共用、共享、共管机制。（国家卫生计生委、国家发展改革委负责）
　　加强卫生应急体系建设。依托现有机构，布局建设国家紧急医学救援基地和区域紧急医学救援中心，构建陆海空立体化的紧急医学救援网络，完善核辐射和中毒紧急医学救援网络，切实提升重特大突发事件的紧急医学救援水平。提高突发急性传染病医疗救治能力。加强县乡两级急救体系建设。（国家卫生计生委、国家发展改革委、中央军委后勤保障部卫生局负责）
　　加强基层医疗卫生机构服务能力建设。以贫困地区为重点，加强乡镇卫生院、社区卫生服务机构标准化建设，提升基层医疗卫生服务能力和水平。推进乡镇卫生院和村卫生室一体化管理。每千常住人口基层医疗卫生机构床位数达到1.2张，重点加强护理、康复病床的设置。（国家卫生计生委负责）
　　加强专业公共卫生机构能力建设。加强疾病预防控制机构建设，实现每个省级疾病预防控制机构内有1个达到生物安全三级水平的实验室，有需要的地市级和县级疾病预防控制机构内有1个达到生物安全二级水平的实验室。建设完善检验检疫系统生物安全三级水平的实验室。提高精神专科服务能力。全面改善妇幼保健和计划生育服务机构的基础设施条件。提升妇幼健康服务机构在孕产保健、出生缺陷防治、儿童保健、妇女保健、计划生育方面的技术与服务能力。加强职业病防治能力、卫生计生综合监督执法能力和食品安全技术支持体系建设。加快改善血站业务用房条件。（国家卫生计生委、国家发展改革委、质检总局负责）

专栏9　卫生计生服务体系建设工程

贫困地区基层服务能力建设：以贫困地区为重点，支持县级医院（含中医医院）业务用房建设，推动乡镇卫生院建设，基层医疗卫生机构标准化达标率达到95%以上，打造30分钟基层医疗服务圈。（国家卫生计生委、国家发展改革委负责）

妇幼健康和计划生育服务能力建设：强化孕产妇和新生儿危急重症救治能力建设，支持省、地市、县三级妇幼健康服务机构服务能力建设，全面改善妇幼健康服务条件，新增产床8.9万张。（国家卫生计生委、国家发展改革委负责）

公共卫生服务能力建设：增强应急能力，依托现有机构建设国家移动应急平台和省级核辐射医疗救治基地。加强省级、地市级、县级疾病预防控制机构业务用房建设。加强省级职业病、传染病、地方病、结核病等防治机构基础设施建设。支持省级血液中心、地市级中心血站业务用房建设。（国家卫生计生委、国家发展改革委负责）

疑难病症诊治能力建设：在肿瘤、心脑血管、呼吸等薄弱领域，支持省部级综合或专科医院建设。（国家卫生计生委、国家发展改革委负责）

　　（十二）加强人才队伍建设。
　　优化人才队伍的规模与结构。医护比达到1∶1.25，市办及以上医院床护比不低于1∶0.6，每千常住人口公共卫生人员数达到0.83人，人才规模与我国人民群众健康服务需求相适应，城乡和区域医药卫生人才分布趋于合理，各类人才队伍统筹协调发展。（国家卫生计生委负责）
　　完善人才培养体系。加强医教协同，建立医学人才培养与卫生计生行业人才需求相适应的供需平衡机制，加强对医学院校设置、区域布局以及医学专业学科结构、学历层次、招生规模的宏观调控，增加人才短缺省份毕业生供给。支持有条件的高校增设儿科学、精神医学本科专业，支持高校根据行业需求合理确定儿科学、精神医学本科专业招生规模。加大对中西部地区高等医学院校的支持，缩小区域、院校和学科专业之间培养水平的差距。完善毕业后医学教育制度。全面实施住院医师规范化培训制度，扩大招收规模，重点向全科和儿科、精神科等急需紧缺专业倾斜，到2020年所有新进医疗岗位的临床医师均接受住院医师规范化培训。逐步建立专科医师规范化培训制度。加强培训基地和师资队伍建设。巩固完善继续医学教育制度，建设一批继续医学教育基地，全面提升各级各类卫生计生人员的职业综合素质和专业服务能力。基本建成院校教育、毕业后教育、继续教育三阶段有机衔接的标准化、规范化临床医学人才培养体系。院校教育质量显著提高，毕业后教育得到普及，继续教育实现全覆盖。（国家卫生计生委、教育部、财政部、人力资源社会保障部、国家中医药局负责）
　　加大人才培养力度。推进以全科医生为重点的基层医疗卫生队伍建设。制订优惠政策，为农村订单定向免费培养医学生。启动实施助理全科医生培训。继续实施基层医疗卫生机构全科医生特设岗位计划，优先安排特岗全科医生到集中连片特困地区乡镇卫生院工作。加强产科、儿科、精神、老年医学、药学、护理、急救、康复等各类紧缺人才以及生殖健康咨询师、护理员等技能型健康服务人才培养。加强高层次人才和公共卫生专业人才队伍建设。加强医院院长职业化培训。加强乡村医生队伍建设。（国家卫生计生委、教育部、财政部、人力资源社会保障部、国家中医药局负责）
　　创新人才使用、管理和评价机制。健全以聘用制度和岗位管理制度为重点的事业单位用人机制。建立符合医疗行业特点的人事薪酬制度，着力体现医务人员技术劳务价值，优化医务人员职业发展环境。健全基层及紧缺人才激励与约束机制，基层医疗卫生机构内部分配要向关键岗位、业务骨干和作出突出成绩的工作人员倾斜，缩小不同层级医疗卫生机构之间实际收入的差距。落实基层卫生专业技术人员职称评审政策，建立符合基层医疗卫生工作实际的人才评价机制。通过人才服务一体化、柔性引进等多种方式，建立完善城乡联动的人才管理和服务模式。创新公立医院机构编制管理方式，完善编制管理办法，积极探索开展公立医院编制管理改革试点，落实公立医院用人自主权。随着经济社会发展，逐步提高乡村医生待遇水平，完善乡村医生养老政策，稳定和优化村医队伍。（国家卫生计生委、人力资源社会保障部、中央编办、财政部负责）

专栏10　人才发展项目

以全科医生为重点的基层卫生计生人才能力建设：通过开展全科专业住院医师规范化培训、助理全科医生培训、全科医生转岗培训、农村订单定向医学生免费培养，培养培训全科医生。培训全科医学师资。加强城乡基层医疗卫生机构骨干人才培训。（国家卫生计生委、教育部、人力资源社会保障部、国家中医药局负责）

医师规范化培训：规范化培训住院医师50万名。加强规范化培训基地建设。启动专科医师规范化培训试点。培训住院医师师资。（国家卫生计生委、人力资源社会保障部、国家中医药局负责）

县级骨干医师培训：以儿科、妇产科、精神科、病理、康复、老年医学、院前急救等为重点，培训县级医院临床骨干医师，全面提高县级医院服务能力和水平。（国家卫生计生委负责）

完善生育政策服务人才保障：加大妇幼健康领域专业人才培养力度。广泛开展产科、儿科医务人员岗位培训和转岗培训，采取多种形式力争增加产科医生和助产士14万名。（国家卫生计生委负责）

医药卫生创新人才队伍建设：吸引、遴选和造就一批具有国际领先水平的医学领军人才，培养、造就新一代杰出中青年学术带头人，吸引、稳定和培养一批有志于医疗卫生事业的优秀青年骨干人才。支持国家优先发展学科和国际科技前沿领域优秀创新团队。（国家卫生计生委负责）

　　（十三）加强人口健康信息化建设。
　　促进人口健康信息互通共享。依托区域人口健康信息平台，实现电子健康档案和电子病历的连续记录以及不同级别、不同类别医疗机构之间的信息共享。全员人口信息、电子健康档案和电子病历三大数据库实现数据融合、动态交互和共享，基本覆盖全国人口并实现信息动态更新。建成统一权威、互联互通的国家、省级、地市级、县级人口健康信息平台，实现公共卫生、计划生育、医疗服务、医疗保障、药品供应、综合管理等六大业务应用系统的资源共享和业务协同。普及应用居民健康卡，积极推进居民健康卡与社会保障卡等公共服务卡的应用集成，实现居民健康管理和医疗服务一卡通用。依托国家电子政务网和政府数据共享交换平台，实现各级平台和各级各类卫生计生机构的互联互通和信息共享。建立完善人口健康信息化标准规范体系，强化标准规范的建设和应用管理。面向在线医疗健康信息服务，实施网络安全战略，加强信息安全防护体系建设。引导自主可控的标准化信息产品研制与应用。（国家卫生计生委、国家发展改革委、中央网信办、工业和信息化部、人力资源社会保障部负责）
　　积极推动健康医疗信息化新业态快速有序发展。全面实施“互联网＋”健康医疗益民服务，发展面向中西部和基层的远程医疗和线上线下相结合的智慧医疗，促进云计算、大数据、物联网、移动互联网、虚拟现实等信息技术与健康服务的深度融合，提升健康信息服务能力。鼓励建立区域远程医疗业务平台，推动优质医疗资源纵向流动，远程医疗服务覆盖50%以上的县（区、市）。全面深化健康医疗大数据应用。推进健康医疗行业治理、临床和科研、公共卫生大数据应用，加强健康医疗数据安全保障和患者隐私保护，积极应用物联网技术、可穿戴设备等，探索健康服务新模式，发展智慧健康医疗便民惠民服务，强化预防、治疗、康复的精细服务和居民连续的健康信息管理业务协同，提高服务能力和管理水平。积极发展疾病管理、居民健康管理等网络业务应用，推进网上预约、线上支付、在线随访、健康咨询和检查检验结果在线查询等服务。以居民电子健康档案为基础，整合居民健康管理及医疗信息资源，开展居民医疗健康信息服务，提高居民自我健康管理能力。完善统计制度，加强统计数据分析能力。（国家卫生计生委、国家发展改革委、中央网信办、工业和信息化部负责）

专栏11　人口健康信息化建设工程

人口健康信息化建设：按照填平补齐、查缺补漏的原则，完善省级、地市级、县级平台，实现省级与国家级平台对接，省内平台互联互通和业务协同。巩固完善传染病防控、预防接种、严重精神障碍等报告与管理工作，突出电子病历与电子健康档案信息动态更新。开展大数据和远程医疗应用试点。推进健康医疗大数据应用，加强区域临床医学健康数据示范工作，推动远程会诊、远程诊断（影像、病理、心电）、预约诊疗、双向转诊等惠民服务。（国家卫生计生委、国家发展改革委、中央网信办负责）

　　（十四）加强医学科技创新体系建设。
　　全面推进卫生与健康科技创新。围绕恶性肿瘤、心脑血管等重大疾病及罕见病等健康问题和健康产业发展需求，加强医学科学前沿基础研究、关键技术研发、成果转移转化、医药产品开发和适宜技术推广。启动实施面向2030年的健康保障重大工程，继续组织实施“重大新药创制”和“艾滋病和病毒性肝炎等重大传染病防治”两个国家科技重大专项，组织实施“精准医学研究”等一批国家重点研发计划，加快诊疗新技术、药品和医疗器械的研发和产业化，显著提高重大疾病防治和健康产业发展的科技支撑能力。加强转化医学国家重大科技基础设施、国家临床医学研究中心和协同研究网络建设，推动现有若干国家重点实验室等国家科研基地的能力提升，调整和完善委级重点实验室，逐步构建规范、整合、高效的医学科技基地平台体系。加强医学科技创新政策环境建设，健全创新人才培养、新技术评估、医学研究标准与规范、医学伦理与科研诚信、知识产权等保障机制，大幅提升医学科技成果转移转化率。发挥国家临床医学研究中心和协同研究网络的作用，促进适宜技术、诊疗指南和技术规范的普及推广。（科技部、国家卫生计生委、国家发展改革委负责）

专栏12　健康科技项目

健康科技重大项目和工程：健康保障重大工程，国家科技重大专项“重大新药创制”和“艾滋病和病毒性肝炎等重大传染病防治”专项；国家重点研发计划“精准医学研究”、“重大慢性非传染性疾病防控研究”、“生殖健康及重大出生缺陷防控研究”等重点专项；中国医学科学院医学与健康科技创新工程。（科技部、国家卫生计生委负责）

成果转化和适宜技术推广：健康科技成果转移转化行动，基层医疗卫生服务适宜技术推广。（科技部、国家卫生计生委负责）

　　**四、**保障措施
　　（一）全面深化医药卫生体制改革。实行医疗、医保、医药联动改革，建立健全覆盖城乡居民的基本医疗卫生制度。健全全民医疗保障制度。加强城乡居民大病保险、重特大疾病医疗救助工作，完善疾病应急救助制度。健全基本医保稳定可持续筹资和报销比例调整机制。整合城乡居民基本医保政策和经办管理。加快推进基本医保异地就医直接结算。全面推进公立医院综合改革。建立现代医院管理制度，完善医院法人治理机制和外部监管机制。控制医药费用不合理增长。健全药品供应保障机制，完善国家药物政策体系，巩固完善基本药物制度，建立药物临床综合评价体系，加强儿童、老年人等特殊人群基本用药保障。推进基本公共卫生服务均等化，完善国家基本公共卫生服务项目，继续实施国家重大公共卫生服务项目。巩固完善基层运行新机制。（国务院医改办、国家卫生计生委、国家发展改革委、财政部、人力资源社会保障部、食品药品监管总局、民政部等相关部门负责）
　　（二）建立公平有效可持续的筹资体系。进一步明确政府、社会与个人的卫生计生投入责任，完善合理分担机制，缓解个人就医经济负担。明确政府在提供公共卫生和基本医疗服务中的主导地位，加大政府卫生投入，保障人民群众的基本医疗卫生服务需求。鼓励和引导社会力量加大对卫生与健康事业的投入，形成投资主体多元化、投资方式多样化的办医体制。（国家卫生计生委、财政部、人力资源社会保障部负责）
　　（三）完善卫生计生法制体系。推动基本医疗卫生法立法工作。完善卫生计生法律法规体系，加强医疗、医药、医保、公共卫生、计划生育等重点领域法律法规的制修订工作。做好部门规章立改废释。加强规范性文件的合法性审查，健全依法决策机制。定期开展规章规范性文件清理和标准复审，维护医药卫生法律法规体系的协调性、一致性。健全卫生标准体系，促进强制性卫生标准的实施。深化行政审批制度改革，进一步推进简政放权、放管结合、优化服务改革，创新卫生计生行政管理方式，加快政府职能转变。推进行政审批规范化建设，严禁对已经取消的行政审批事项继续和变相审批，加强承接机构能力建设，确保取消下放事项落实到位。推进政务公开。（国务院法制办、国家卫生计生委、人力资源社会保障部、食品药品监管总局负责）
　　（四）强化宣传引导。加强正面宣传和典型宣传，增强社会对健康和卫生计生工作的普遍认知，争取各方面的有力支持，保障规划的有效实施。加强社会宣传工作，通过电视、广播、报纸和网络等媒体广泛宣传健康和卫生计生工作相关法律法规和面临的形势与挑战，提高社会各界对健康和卫生计生工作的重视程度。加强卫生计生普法宣传。大力弘扬和践行卫生计生职业精神，深入开展职业精神宣传推介专题活动。完善新闻发布制度和网上舆论工作体系，及时回应网上舆情和社会关切，加强网络舆论引导队伍建设，提升新闻宣传与舆论引导能力。发展健康文化，加强卫生计生文化建设和精神文明建设，建设卫生计生文化宣传基地和文化推广平台。（中央宣传部、国家卫生计生委、中央网信办负责）
　　（五）做好国际交流合作。制订中国全球卫生战略，实施适应不同国家、地区和组织特点的多层次、多渠道合作策略，提升我国在全球卫生外交中的影响力和国际话语权。积极推进“一带一路”建设中的卫生交流与合作。加强2030年可持续发展议程、全球卫生、医药卫生科研、人口与发展等领域的合作，引进卫生计生改革与发展所需的智力、技术等资源。创新工作模式，继续加强卫生援外工作。推进全球卫生人才培养和队伍建设。深化与港澳台地区的医疗卫生合作交流。推进南南合作。推动医疗设备和药品“走出去”。大力发展国际医疗健康服务贸易，加强中医药国际交流与合作。（国家卫生计生委、国家中医药局、国家发展改革委、商务部、食品药品监管总局负责）
　　（六）加强组织实施。各级政府要从全面建成小康社会、推进健康中国建设的高度，进一步提高认识，加强领导，将卫生与健康工作纳入重要议事日程。各有关部门要按照职责分工，细化目标，做好相关任务的实施工作。逐步建立健康影响评价评估制度。建立健全监测评价机制，国家卫生计生委负责牵头制订规划监测评估方案，并对规划实施进度和效果进行年度监测和中期、末期评估，监督重大项目的执行情况，及时发现实施中存在的问题，并研究解决对策。地方各级人民政府要定期组织对当地规划实施情况的检查督导，确保规划顺利实施。（国家卫生计生委牵头）

© 北大法宝：（[www.pkulaw.com](https://www.pkulaw.com/)) 专业提供法律信息、法学知识和法律软件领域各类解决方案。北大法宝为您提供丰富的参考资料，正式引用法规条文时请与标准文本核对。
欢迎查看所有[产品和服务](https://www.pkulaw.com/helps/2.html)。法宝快讯：[如何快速找到您需要的检索结果？](https://www.pkulaw.com/helps/70.html)[法宝](https://www.pkulaw.com/helps/69.html)V6有何新特色？

扫描二维码阅读原文

**原文链接：**<https://www.pkulaw.com/chl/dbda350786108bbcbdfb.html>
